# Towards Quantitative Optical Cross Sections in Entomological Laser Radar – Potential of Temporal and Spherical Parameterizations for Identifying Atmospheric Fauna

**DOI:** 10.1371/journal.pone.0135231

**Published:** 2015-08-21

**Authors:** Mikkel Brydegaard

**Affiliations:** 1 Lund Laser Center, Department of Physics, Lund University, Sölvegatan 14, SE 223 62, Lund, Sweden; 2 Centre for Animal Movement Research, Department of Biology, Lund University, Sölvegatan 37, SE 223 62, Lund, Sweden; 3 Hyspex, Norsk Elektro Optikk A/S, Prost Stabels Vei 22, N-2019, Skedsmokorset, Norway; University of Tours, FRANCE

## Abstract

In recent years, the field of remote sensing of birds and insects in the atmosphere (the aerial fauna) has advanced considerably, and modern electro-optic methods now allow the assessment of the abundance and fluxes of pests and beneficials on a landscape scale. These techniques have the potential to significantly increase our understanding of, and ability to quantify and manage, the ecological environment. This paper presents a concept whereby laser radar observations of atmospheric fauna can be parameterized and table values for absolute cross sections can be catalogued to allow for the study of focal species such as disease vectors and pests. Wing-beat oscillations are parameterized with a discrete set of harmonics and the spherical scatter function is parameterized by a reduced set of symmetrical spherical harmonics. A first order spherical model for insect scatter is presented and supported experimentally, showing angular dependence of wing beat harmonic content. The presented method promises to give insights into the flight heading directions of species in the atmosphere and has the potential to shed light onto the km-range spread of pests and disease vectors.

## Introduction

The atmospheric fauna comprises birds, bats and insects and has fascinated biologists for centuries. Insects are the most diverse animal group on earth, are important for ecosystem services[1], and can be major agricultural pests and disease vectors[2]. Whereas a sustainable population of pollinators is crucial for food production and survival of mankind, pests such as migratory moths and locusts can have a devastating effect on agriculture or forestry. Furthermore, mosquitoes and biting midges can transmit deadly diseases to humans and livestock[3,4]. The atmospheric insect fauna can also serve as an indicator of the diversity and functioning of ecosystems. Techniques that enable monitoring of insects can hence provide information of importance for the management of disease vectors and pest species and additionally a method for evaluating ecosystem functioning.

Despite technological advances including a number of advanced tagging and tracking devices successfully applied in the field of ornithology and entomology, it remains exceedingly difficult to study the smallest animals present in the atmosphere. Various kinds of insect traps may provide precise species, gender, age classification, micromorphology, dietary information through, e.g. isotope mass spectrometry and genetic analysis. At the same time, many trapping methods are known to be biased and perturbing[5], thus continuous online non-perturbing *in situ* surveillance is a much desired complimentary tool, and constantly increasing specificity remains on the entomologist’s wish list. There are no gold standards in determining *in-situ* abundances, population sizes, landscape dispersal rates or global fluxes of flying insects. Whereas the trophic interactions between larger animals can often be observed visually, aerial interactions between insects require a tremendous technical efforts to document[6], and it is challenging to make the focal species behave as in their natural habitat. Direct assessment of *in-situ* multi-species interaction strengths governing the population dynamics are exceedingly hard to achieve. This could be one future application of entomological laser radar (lidar).

Radar has revolutionized ornithology in the past century[7], and the experimental possibilities of radar entomology have recently been outlined in a book covering half a century’s work[8]. Most insects are, however, typically too small to be resolved individually with radar, and lidar could revolutionize the understanding of flying insects in the decades to come. Our group is currently pushing sensitivity, specificity and other factors beyond previously applied techniques in the remote sensing of insects. Atmospheric lidars are sensitive enough to retrieve the reflectance from entirely clean air, any addition to the air volume, such as insect, would further increase the backscatter. In general, there is no fundamental nor technical detection limits for either altitude nor for organism size in entomological lidar; system costs would set the main constraints. The range resolution can be as small as a few cm and sampling rates in several kHz. The main advantage of lidars over radars is that systems are easily reconfigured in terms of spectral bands, polarization modes, or quadrant detection with off-the-shelf components[9]. Further, the spectral information in the optical regime can be associated with specific molecules such as melanin, wax, chitin or hæmoglobin and also microstructures such as wing membrane thickness or feather barbule periodicity[10]. Evaluating the de-polarization ratio[11] in the optical regime would provide information on, for example, body furriness or wing glossiness. Whereas large insects can be tagged with electrical diodes in radar entomology[12,13] corresponding tagging can be accomplished by less perturbing fluorescent powders in lidar entomology[14]. Lidars are not capable of penetrating clouds like radars are. Most atmospheric lidar activity requires extensive consideration in respect to public eye-safety, whereas this is not a consideration with radar.

Atmospheric lidars[15] have been used for several decades in the aerosol community [16], for the classification, quantification and investigation of smog, soot, mineral dust, sea salt, pollen as well as bio-warfare agents[17]. Most such lidar systems operate at slow repetition rates of 10–20 Hz and the community presupposes that the atmosphere can be assumed essentially static above few tens of Hertz. In comparison we have observed frequencies associated with insect wing beat frequency (WBF) up to 10 kHz, and typically see transit times through our probe volume down to 10 ms. The latter would require sample rates beyond 100 Hz in order to observe the same individual in two consecutive shorts. Consequently, conventional lidars incorporate so-called “single photon counting” electronics regardless of whether the assumed “single photons” are encountered simultaneously in all wavelength- and polarization channels, which would be the case in aerofauna observations. Although atmospheric fauna events in lidar data can be considered rare [18] in respect to not observing an event, our group has recently reached in the order of 10^4^ events/(hour m^3^). Such numbers severely change the back-scatter probability distribution [19], introducing skewness which is absent in molecular and aerosol return (which can be represented by Gaussian noise about a static value).

**Fig 1:**
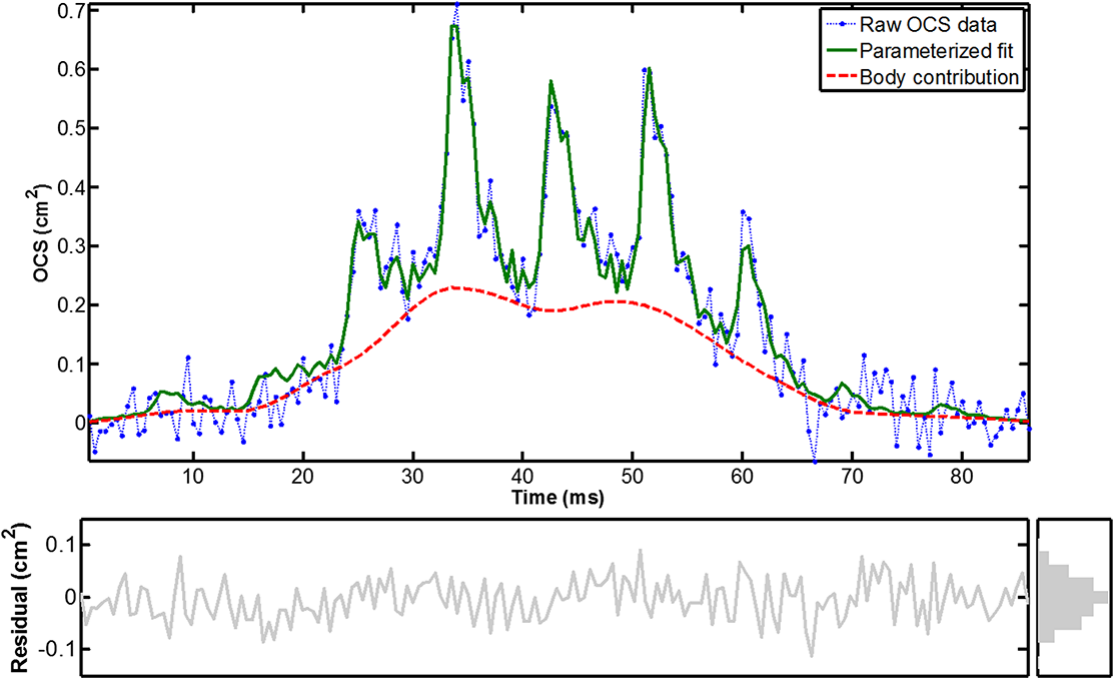
Representative example of an insect detection event observed with the LUMBO lidar, from a distance of one km at 2kHz sample rate and 808 nm wavelength. The parametrized fit with discrete harmonics describes the raw data fairly. Judging from a wing beat frequency of 113 Hz, night time observation, and that the waveform is not spiky as for glossy wings it is presumably a moth. While many local moth species are rather bright the true projected cross section could be roughly twice of the OCS value due a reflectance of ~50%.

Due to the limitations of conventional atmospheric lidar systems, we have recently developed a continuous wave (CW) kHz lidar method [9], which is implemented in the Lund University Mobile Biosphere Observatory (LUMBO)[20,21] and also in smaller systems[22] in the process of commercialization. The system is currently constructed with a 3W, 808 nm laser diode and has been operated up to 5 kHz sample rates with insect observations at several km range; see Fig 1 (S1 File). Entomological lidars have also previously been developed using kHz sampling rates by another group [23]. Although some values for radar cross sections and fundamental wing beat frequency have been determined[8], there are an estimated several million insect species worldwide, each with sex and several age groups, as well as some which can fly tandem, carry pollen, eggs or blood meals.

A key to success of lidar entomology is therefore to produce quantitative values and look-up tables for the optical cross section (OCS) of species, genders and age groups which can be used across studies. Although it would be unrealistic to determine OCS tables for every single species, sex, age and payload worldwide, priority could be given to tabulate major classes and species of special interest, e.g., such as known bioindicators, important pollinators, agriculture- and forestry pests, and vectors for diseases such as malaria and blue tongue fever. Even a library with cross sections for a smaller set of species would greatly increase specificity and allow the interpolation between table values for a variety of species for the purpose of qualified guessing non-tabulated species.

One major obstacle for producing table values for the OCS of insects is that the OCS varies as a function of phase in the wing beat cycle as well as with the observation aspect or angles in respect to the anatomical coordinate system of the individual. The present work aims to outline how table values of insect OCS can be expressed employing well-known methods from, e.g., atomic physics.

## Light Scattering from Insects

A number of aspects of spectroscopy and light interaction with insects have been elaborated on in a remote sensing context [24]. More recently our group have made efforts to calibrate the echo strength to backscatter OCS and extinction OCS in the unit of, e.g., cm^2^. In lidar experiments this can be achieved by terminating the beam on a hard dark target with a known diffuse reflectance, this provides a fix-point for OCS on the range resolved echo. The system sensitivity is then extrapolated back towards the lidar by using the static molecular return after verifying that the atmosphere is homogeneous. The procedure is described in details in Ref. [25]. In cases where the probe volume is accessible the OCS calibration can be additionally verified by weaving a stick with known OCS or dropping Teflon spheres through the probe volume at various distances[26]. In dark-field experiments [27] and in laboratory, the calibration is done with Teflon spheres in various sizes. It is assumed that the spheres are 100% opaque and have a Lambertian reflectance of 100%. The resulting OCS values for insects thus equal their projected area had they been diffuse white. The world of insect can be incredible colorful and mechanism for manipulating light can be very sophisticated[28], but a very crude description for insects in general[29] is that reflectance in the visible (VIS) region is in the order of 5%, in the near infrared (NIR) reflectance in the order of 20%, and in the short wave infrared (SWIR) reflectance peaks at some 80% for most insects. Specular reflectance can reach values as high as 1000% compared to a diffuse white target, therefore mainly the diffuse or de-polarized backscatter as well as extinction represents the actual projected size (extinction is often very challenging to retrieve by lidar systems). A unique property of a flying insect is that its OCS oscillates. Lidar observation of atmospheric fauna such as the event presented in Fig 1 can be parameterized by a discrete set of harmonics[30] (see green line in Fig 1), as in Eq 1:

$$OCS\left(t\right)=\beta \left(t\right)\;\sum_{h=0}^{h<½f_{s}}\left(c_{1,h}\text{sin}\left(2\pi f_{0}ht\right)+c_{2,h}\text{cos}\left(2\pi f_{0}ht\right)\right)$$ (1)

Here, *t* is time, and the weighting *β* is a time series corresponding to the non-oscillatory body scatter contribution (see red line in Fig 1). This is obtained from low-pass filtering with a blocking frequency corresponding to the fundamental WBF. This weighting separates the observation envelope from the intrinsic modulation by the insect. *h* is the running integer index of the harmonic, *f*_*s*_ the sampling frequency, *f*_*0*_ the fundamental WBF and *c*, the optical cross section coefficients for corresponding harmonics in units of area. Eq 1. relies on the robust estimation of *f*_*0*_ which is not a trivial problem. In ideal cases the beam profile and resulting envelope, *β*, is tophat with the consequence that *β* exhibits side lobes in the modulation spectrum. These contributions must be subtracted before fundamental tone is sought for. Furthermore, the magnitude of the fundamental tone is not necessarily stronger than magnitude of the harmonics, thus the median spacing between harmonics yield a more robust measure of *f*_*0*_ than the strongest feature once the contribution from the envelope is subtracted. When data subjected to the parametrization in Eq 1 it is assumed that fundamental tone and harmonic strengths are constant during transit time through the probe volume.

It is well-known that *f*_*0*_ alone is insufficient for identification to species and that *f*_*0*_ varies with ambient temperature[31]. For improved specificity continuous modulation power spectra or cepstra can be obtained from flying insects; however, the peak widths and side lobes of each harmonic overtone are a non-specific observation artifacts and are merely a measure of the insect transit time through the probed air volume. Further, projection of the time series onto a discrete set of harmonics, as in Eq 1, yields superior numerical certainty. Harmonic modulation spectra from insects have been retrieved from backscattered returns from vertical-pointing radars [8] as well as from vertical optical extinction coefficients in laboratory setups [32,33], and species specificity has been demonstrated from the harmonic content. In the *special case* of vertical geometry and zenith observation in respect to the insect anatomy and direction of gravity, the harmonics dependence on flight heading direction is minimal as opposed to the situation during horizontal monitoring. Therefore harmonic content may be evaluated in vertical monitoring without take the aspect into account. In this report a method for defining consistent cross sections in the *general case* of arbitrary observation is proposed.

Accordingly, it is understood that harmonic OCS from insects monitored from an arbitrary angle will provide a projection and hence not a consistent value as long as the flight heading and pitch is undetermined. In a situation where the heading and pitch is known, absolute OCS can be retrieved for species classification. Furthermore, heading-pitch retrieval can provide valuable information for performing 3D flux measurements of insects moving over specific locations. The following sections will discuss how both absolute OCS of insects can be retrieved from arbitrary angles and at the same time provide flight heading directions.

Coherent specular reflections do not generally appear in radar entomology since the wavelength is often longer than the insect size and specular reflections play a minor role for the modulated optical extinction. The specular reflection relates to the refractive index of the wing membrane and the glittering of insects, which, for some species, is associated with insect age. In for instance damselflies, young individuals which have recently emerged from their pupal stage have flexible and glittering wings. Over time the wing membranes stiffen and become increasingly matte[34]. Optical insect aging has been demonstrated in laboratory settings [35,36], although not targeting the refractive index directly. Whereas the lower harmonics represent the diffuse incoherent scatter from the projection of wing shape and flapping, the specular reflection is observed as one to four narrow spikes during the wing beat waveform. It is responsible for the large number of harmonics overtones. Future remote age determination would be a ground-breaking tool for studying population dynamics in ecological entomology. We have previously briefly illustrated how wing membrane thickness can be provided by spectral probing the specular reflection from insect wings [9,29]. The wing membrane thickness has been shown to be species specific [37].

## A Trajectory of a Fruit Fly

**Fig 2:**
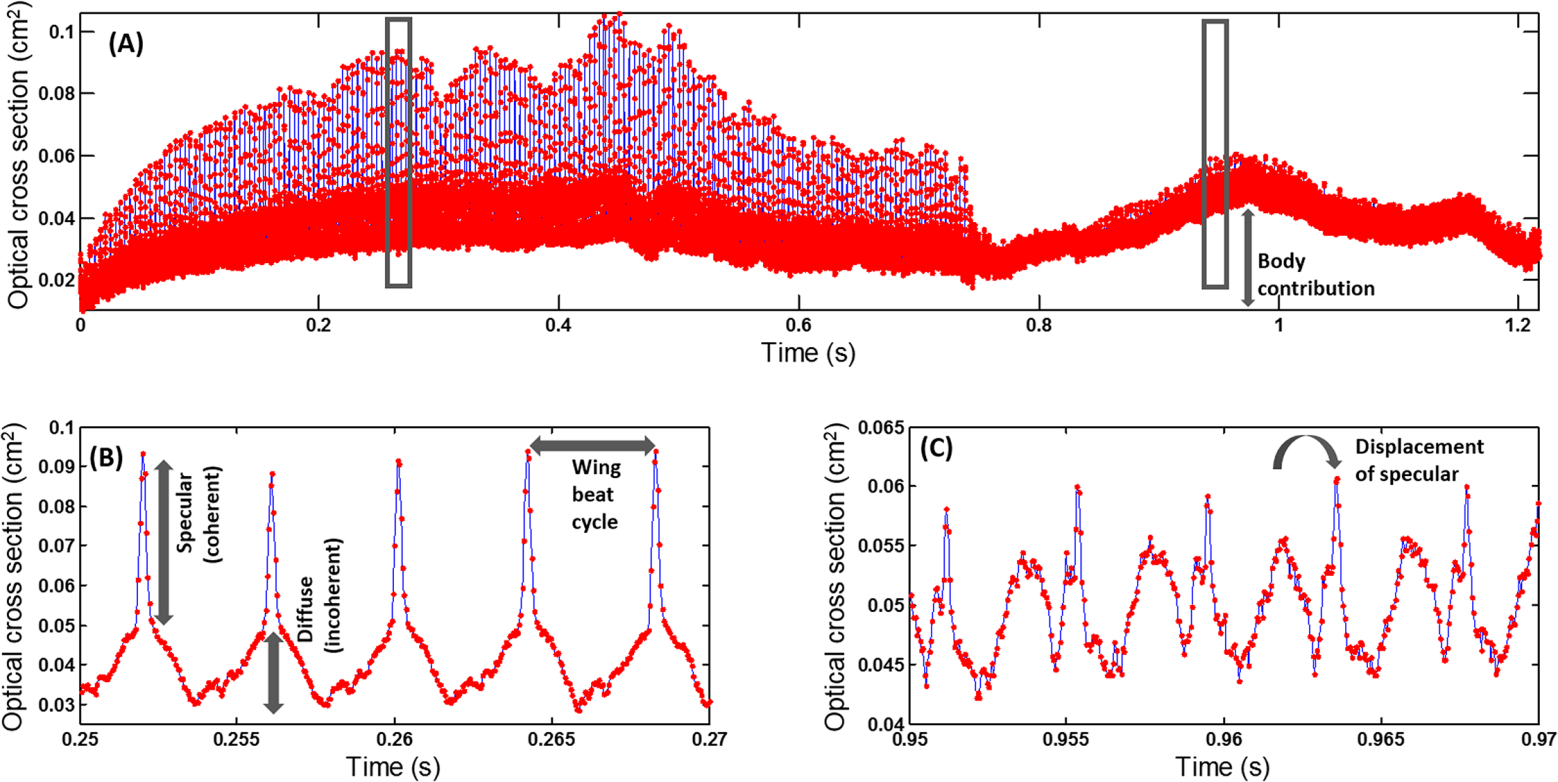
Laboratory recording of backscatter from a common fruit fly (*Drosophila*). (a) shows the entire event whereas zoomed regions in (b) and (c) emphasize the different waveform during the event. Several parameters such as body off-set, specular and diffuse contributions are highlighted in the time series.

**Fig 3:**
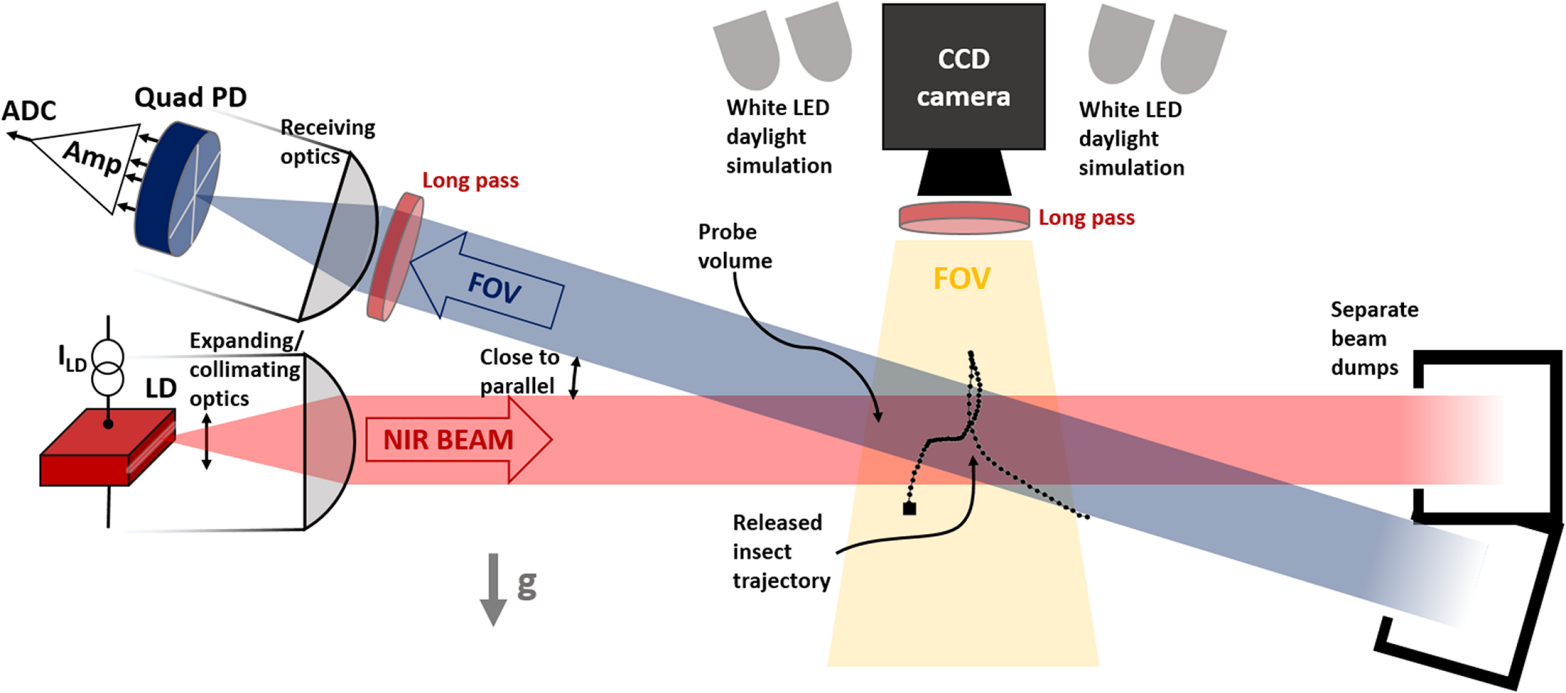
Set-up for backscatter recording. Experimental set-up for simultaneous recording of wing beat harmonics with a quadrant photodiode and flight trajectory with an area-scan CCD camera. White LED illumination gives the specimen daylight perception without interfering with the measurement.

For the purpose of discussing harmonic content from airborne insect targets in relation to flight direction, we will consider a single insect event; see Fig 2 (S1 File),–a fruit fly (*Drosophila melanogaster*, unknown sex) released in a laboratory setup; see Fig 3. The setup comprised a horizontal laser beam and a near-parallel incidence field of view (FOV) of a Si-quadrant diode in close-to-backscatter configuration. An industrial camera and a white GaN LED illumination arrangement were placed in the top part of the release chamber. An 800 mW, 808 nm NIR laser was expanded to ø102 mm and collimated. The beam can be considered to have a top-hat profile and was terminated in a cavity behind the setup. The FOV of the quadrant was expanded and collimated equivalently. The quadrant and trans-impedance amplifier have a bandwidth of approximately 5 kHz, after which modulations are slightly attenuated. The four signals are recorded at 20 kHz sampling rate by a DAQ device (National Instruments USB6211). The quadrant is fitted with an optical long-pass filter (RG780). The signals from the four segments can in principle be separated but in the following section we will consider only the sum of the segments. Quadrant detection in entomological lidar can verify that monitored individuals enters the probe volume entirely, evaluate the focus and give clues on the flight heading direction (this will be discussed later). The overlap between the beam and the FOV was arranged in a chamber where several individuals were kept. The insect entering the beam is monitored from above with an industrial camera (Bassler) also equipped with a long-pass RG780 filter. White LED illumination activates the diurnal fruit flies without perturbing the NIR measurements since the white LED light is blocked by the long pass filter.

**Fig 4:**
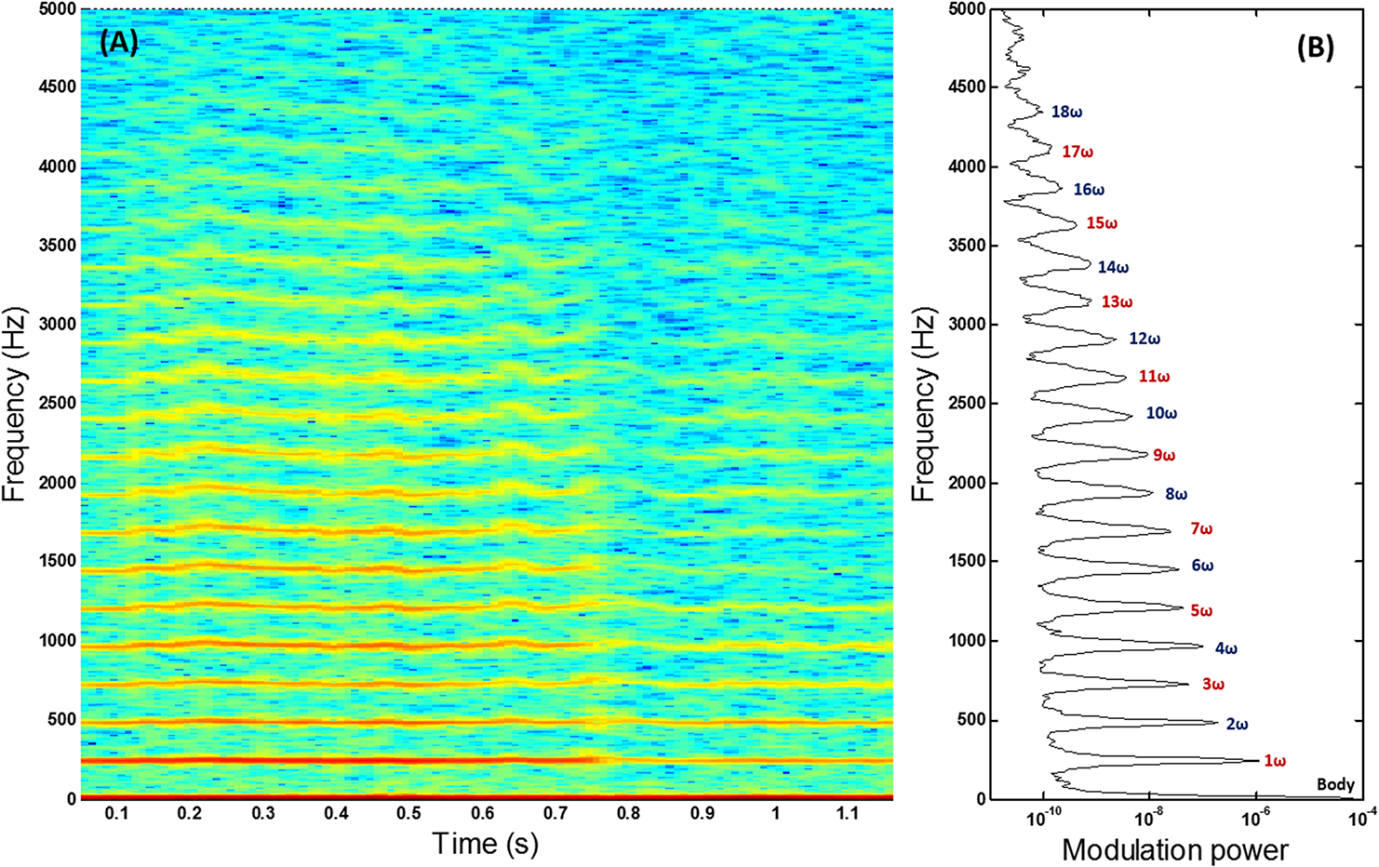
Tempo-spectral analysis. (a) Spectrogram showing the time evolution of the modulation power spectrum retrieved from the fruit fly event. (b) Averaged modulation power spectrum showing at least 18 discernible harmonic overtones.

The individual erratically changes course and speed several times during the intersection with the probe volume. Zoom-in on two intervals during the events reveals entirely different waveforms from the same individual; see Fig 2B and 2C. The fundamental frequency is clearly discernible and fairly stable and the waveform can be summarized through a series of harmonics. Since Eq 1 only treats an observation with constant modulation spectra, the evolution of the harmonics over time is here analysed in a spectrogram; see Fig 4, in order to associate harmonic content with flight heading direction. Due to the specular spikes during the wing beat cycle, a very large number of harmonics is observed.

**Fig 5:**
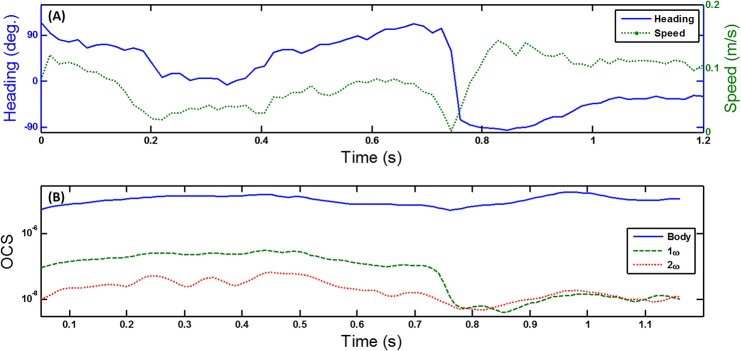
Harmonics in relation to flight heading. (a) Flight heading direction with respect to illumination and observation angle and flight speed, obtained from the perpendicular camera setup. (b) The time evolution of OCS of body, fundamental frequency and second harmonic.

Through image analysis from the sequence from the industrial camera, heading direction and speed is obtained; see Fig 5. A sharp turn of the heading course is observed around t = 0.8 s. Changes in the relative strengths of the body, fundamental and second harmonic OCS coincide with major changes in flight direction and speed.

## First-Order Model for Insect Scattering

**Fig 6:**
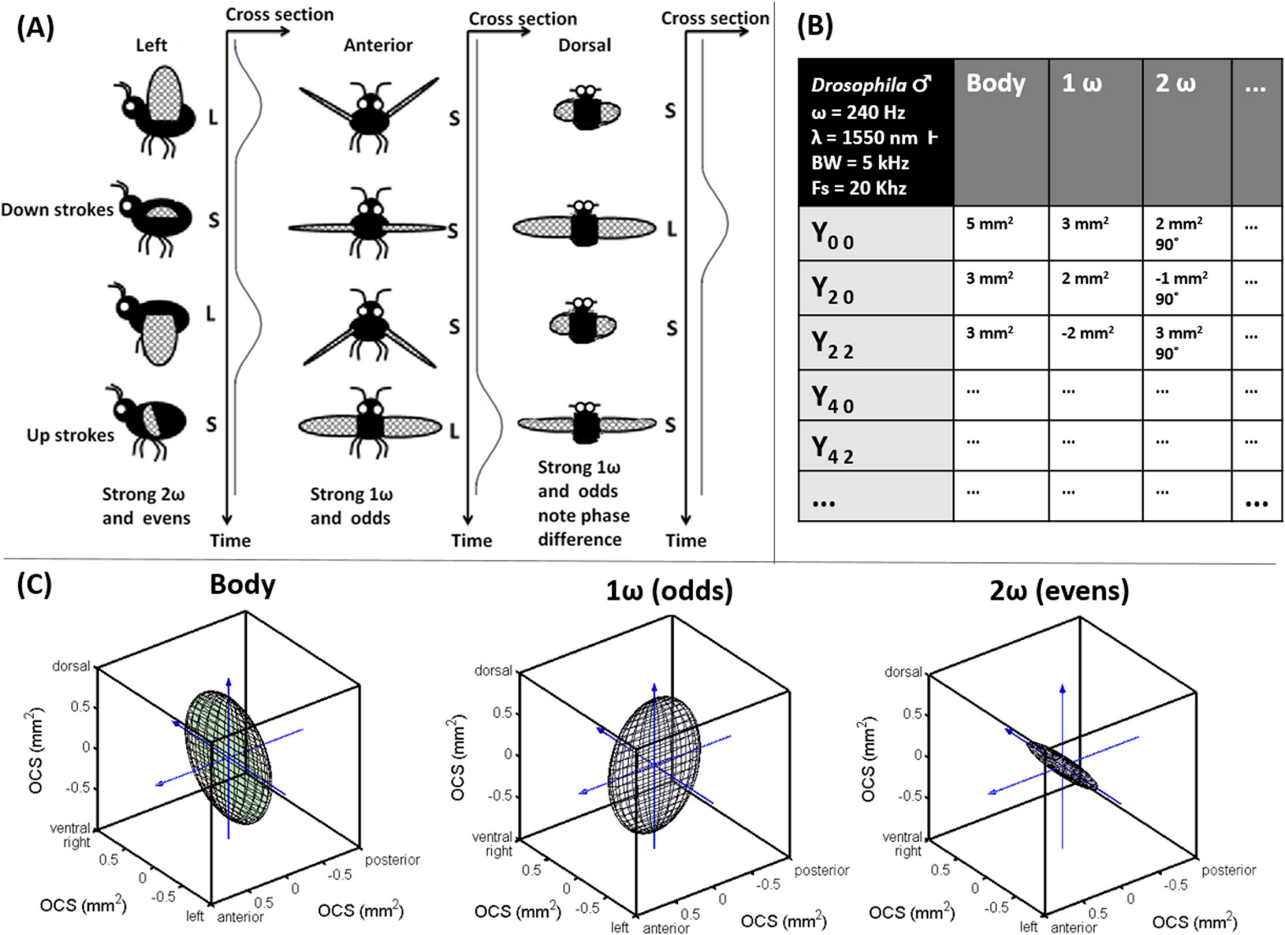
First order model for insect scatter. (a) Basic model for understanding harmonic content in relation to insect anatomy, compare postulates in respect the strength of tones in Fig 1. (b) The author’s idea of table values for insect OCS with speculative values, note the reduced set of harmonics base functions. (c) Visualization of speculative insect OCS, adopted from [26].

For a fundamental understanding of relation of the harmonics to the body orientation, we can consider four phases in the wing beat cycle from the three anatomical planes; see Fig 6A. From the figure and the cross sections over time, the contributions to the body, fundamental frequency and second harmonic are understood.

**Fig 7:**
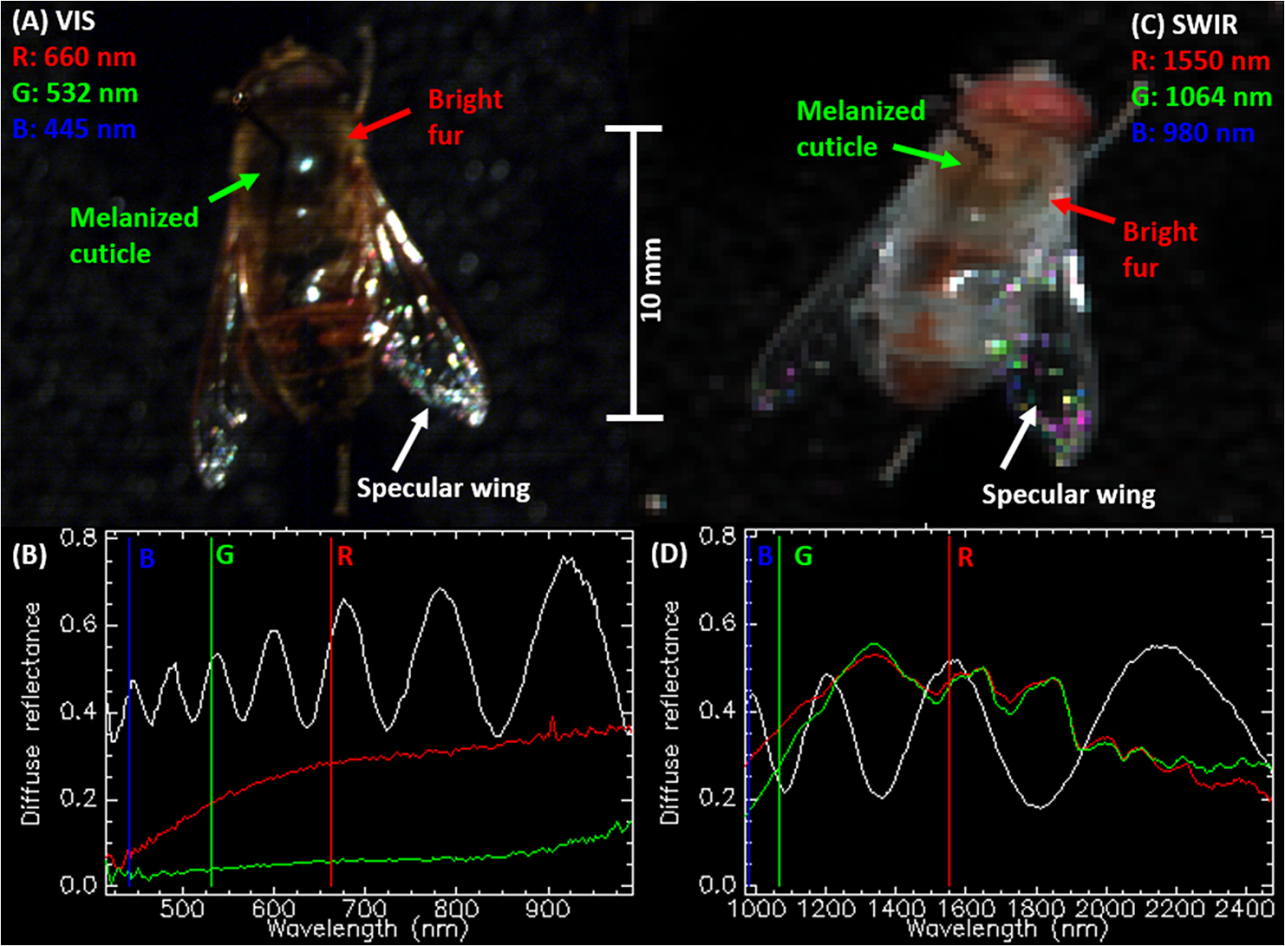
Selected hyper- spectral insect features in the visible and SWIR. (a) True-color picture of a fly. (b) Melanized body part has low reflectance throughout the visible range. (c) False-color SWIR picture of the same fly. (d) Reflectance is identical at 1550 nm regardless of melanization; thus insects are highly symmetrical even in the frontal and transverse plane at this band.

**Fig 8:**
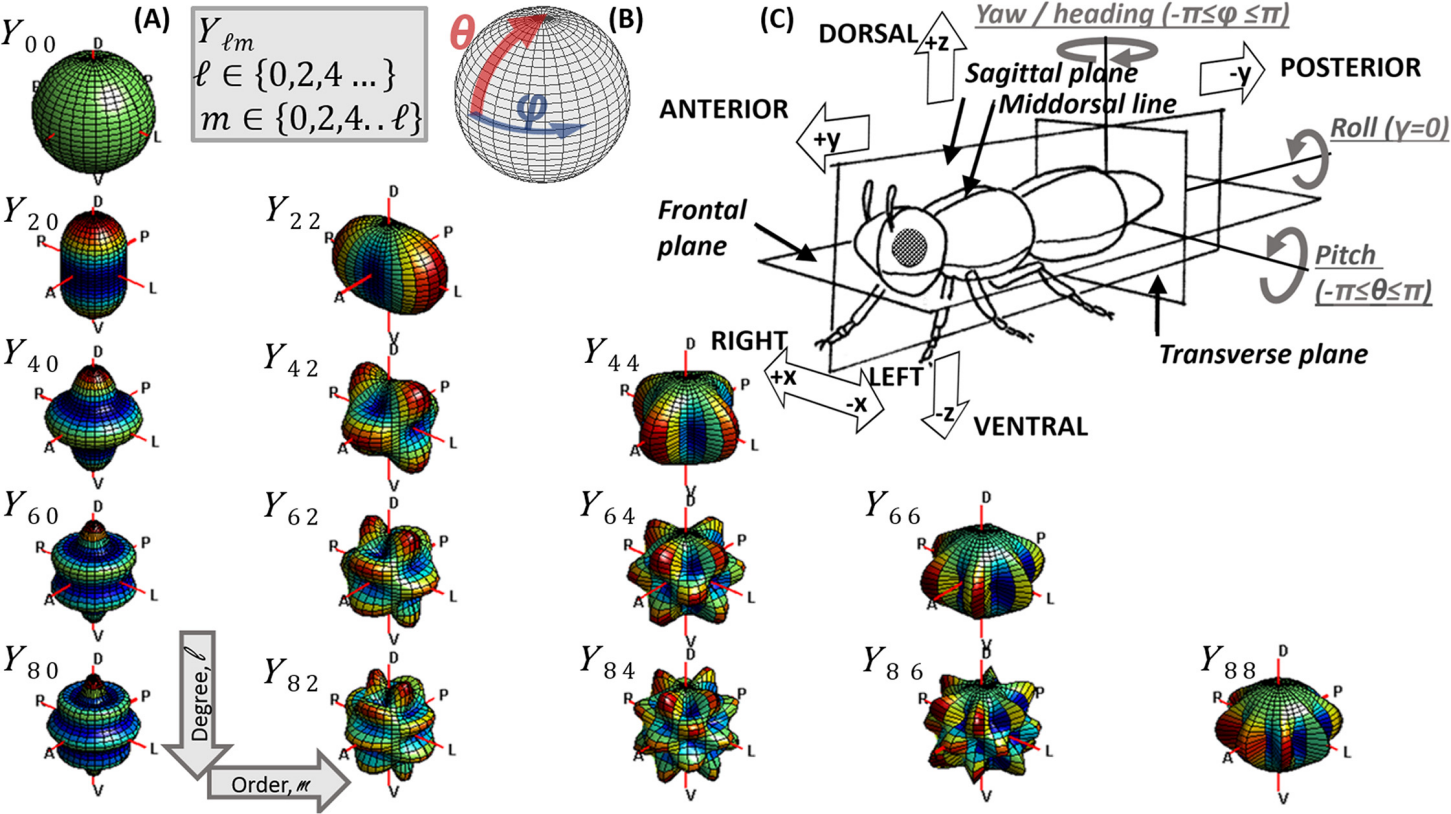
Relations between polar coordinates, spherical harmonics, Cartesian coordinates, anatomy and flight mechanics all seen from the same perspective. (a) Subset of symmetrical spherical harmonics of even degree and order. (b) Polar coordinates, where the polar axis coincides with the dorsal-ventral axis. (c) Anatomical terms and concepts from flight mechanics.

The amount of backscattered (or extinguished) light from an insect at various modulation frequencies, can be described as a spherical functions varying with the heading and elevation angles that the insect intercepts the probe volume (roll angle will be discussed later). These spherical functions are analytical and continuous and can thus be adequately be described by a linear combination of real spherical harmonics. The strengths of the spherical harmonics can be estimated from experimental recordings of OCS in conjunction with synchronized heading and pith angles by regression[38,39]. The spherical scatter function is in general always left-right symmetrical around the mid-sagittal plane. Thus only the subset of real spherical harmonics of even orders are required to express the spherical functions for the body, fundamental and overtones. We have previously described[9,29] how near-infrared wavelengths (NIR), e.g. 808 nm, are affected by melanization. This is again illustrated in Fig 7 (S1 File). Since melanization may differ from anterior to posterior or from ventral to dorsal side, asymmetry can be expected around the frontal- and transverse plane in the NIR. For a short-wave infrared (SWIR) wavelength, 1550 nm, we have shown that the optical cross section essentially is equivalent to the true cross section area for all insects and that OCS in SWIR is insensitive to melanization[9,29]. This implies that insect size can be derived directly from OCS at SWIR and that an absolute measure of the melanization can be derived from the ratio of SWIR/NIR OCS. It also implies that the spherical functions determining SWIR OCS are approximately symmetrical around both frontal, ventral and sagittal plane. This holds true for the diffuse contribution of body as well as the oscillatory wing part. In addition, the specular contribution of the oscillatory part is also three folded symmetric. This because the specular condition is fulfilled when wing surface normal coincides with the midpoint between emitter and receiver of the lidar, and since surface normal is identical on either side of the wings. This may not be the case for the specular contribution from the body DC part. Consequently, SWIR OCS can be represented with an even smaller subset of the complete set of real spherical harmonics, namely only the associated Legendre polynomials with zero or even degree and positive even orders; see Fig 6B and 6C. To remind the reader of polar and anatomical coordinates and symmetric spherical harmonics, an overview is composed in Fig 8.

The various symmetries discussed above imply simplifications in terms of expansions on real spherical harmonics. However, it also implies that heading or pitch angles cannot be determined uniquely, since a directional vector cannot be distinguished from its mirrored counterparts in any of the anatomical symmetry planes. Our approach to break the left-right and up-down symmetry is to employ quadrant detection [9,29]. Here crude flight direction is determined from the consecutive appearance in two of the quadrant segments. We generally always employ quadrant detection, also for the sake of rejecting peripheral observations. The remaining symmetry can be broken by the range time-derivative during insect events [9].

**Fig 9:**
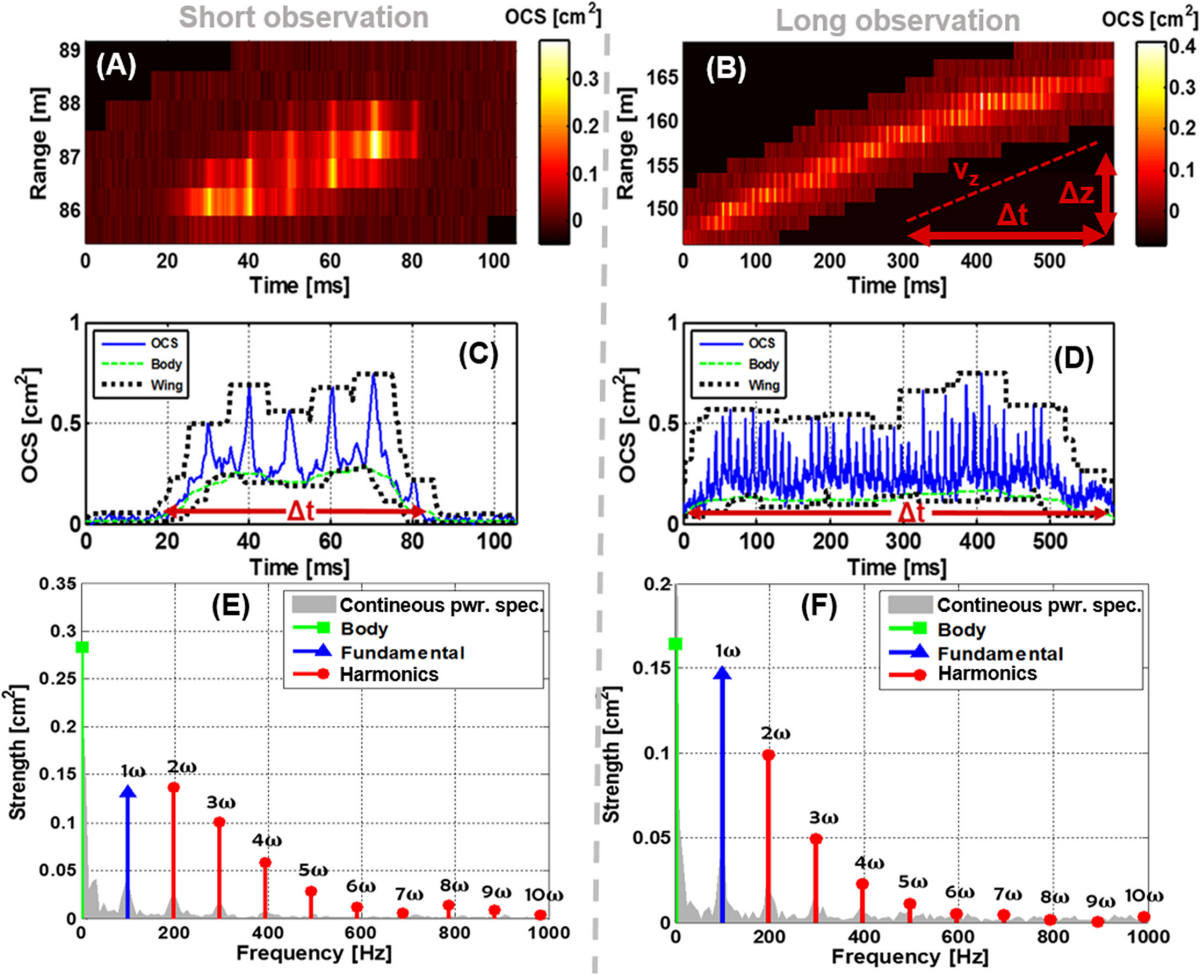
Two entomological lidar observations. (a b) Range-time lidar maps of one short and one long insect observation as encountered in the raw data of our kHz lidars. The range-time derivative indicated provide one component of the velocity. The black mask is added by our algorithms to suppress noise. (c d) The time dependent optical cross section can be decomposed into an oscillatory contribution originating from the wings and a non-oscillatory contributino from the body. (e f) As in Fig 1 the observations can be effectively parametrized by a discrete set of harmonics weighted by the body contribution. The continuous power spectrum is displayed in the background. The two examples have the same fundamental frequency and similar cross sections. Note how the strength of the fundamental tone is weak in respect to body and second harmonic in the case of the short observation and the opposite in the long observation. The duration, range-time derivative and harmonic strengths are coherent with the model presented and the interpretation that Fig 9A is observed close to sagittal plane while Fig 9B is observed close to transverse plane.

In Fig 9A and 9B (S1 File), we present two remote observations and their respective temporal parametrization. The range is closer compared to Fig 1 and consequently SNR is better. The slope in the time-range map indicates the flight velocity along the lidar beam. The two observations both have a fundamental frequency of 100 Hz and comparable cross sections and it is plausible that it is the same species. The transit time, Δt, in the lidar probe volume differs a factor of 10 (Fig 9C and 9D). It is understood from the time-range maps that the individual in Fig 9A intercepts the beam perpendicularly whereas the individual in Fig 9B fly away from the lidar unaware that it is being probed. The parameterization by discrete harmonics of the two cases is presented in Fig 9E and 9D overlaid on the continuous modulation power spectrum. The side lobes and harmonic peak-width in the continuous power spectrum is accounted for by the weighting with the non-oscillatory contribution (this can be considered as a convolution with an envelope). The harmonics strength decays rapidly compared to Fig 4B thus the wings are presumably diffuse compared to the glossy wings of the fruit fly. The strength of the fundamental tone to the strength of both body and second harmonic is in accordance with the understanding and model presented in Fig 6. The proposed behaviors is predominantly for the low harmonics representing the diffuse backscatter.

## Conclusions, Discussion and Perspective

Here, we propose a method for quantitative scatter cross sections for use in remote insect classification and remote surveillance of flying insects over large spatial scales. We have outlined how absolute OCS can be defined and parameterized in time with a discrete set of real harmonic overtones, and we have introduced methods from atomic physics whereupon each temporal overtone is considered as a spherical function which again can be parameterized by a reduced set of real spherical harmonics. For this parameterization, flight heading and pitch is obtained separately by a synchronized camera either in laboratory setup as in Fig 3 or in field[26].

At the common laser wavelength 1550 nm the diffuse or depolarized body backscatter can additionally be described by the spherical harmonics of zero and even order. The depolarized back scatter is roughly equivalent to the incoherent diffuse contribution indicated in Fig 2B. It should be noted that both lidar and laboratory data presented currently do not distinguish co- and de-polarized backscatter although this technically accomplishable. Both diffuse and specular backscatter from wings is expected to be three folded symmetric at SWIR bands. For the application of estimating fluxes and to avoid polar discontinuity we propose to align the polar axis along the ventral-dorsal axis of the insect, where the azimuth heading corresponding order of the Legendre polynomials can only be zero or even at SWIR bands. We have also proposed how to establish table values for the atmospheric fauna for improved lidar specificity and comparability across studies and instrumentation platforms.

Here, only the flight heading angle was acquired by the industrial camera and not the pitch angle. Consequently, the speed in the horizontal plane is recorded with limited precision. For future work we will employ 3D stereo vision with a folding mirror in the cameras’ field of view. This will allow us to retrieve both heading and pitch angles as well as more accurate flight speeds. By means of such procedures, direct predictions of heading and pitch can be attempted and evaluated against the *priori* information from the camera. In analogy with the 18 temporal harmonics discernable in Fig 4B a 3D experiment would yield clues on the number of spherical harmonics required to represent scattering from various species. In general, round body parts and matte wings would be fairly described by a few spherical harmonics. Elongated body parts such as the thorax of dragon flies would require spherical harmonics of high order and degree on the DC component. Specular reflections are expected to yield minute spherical details and corresponding spherical harmonics of very high degree and order. Estimations of heading and pitch angles do not necessarily require such high detail.

It can be argued that the contribution from the specular spikes should be treated differently than the diffuse contribution. Specular reflections could either be distinguished post-processing as fast features in the waveform (high harmonics), or they could also be physically separated by implementing a co- and de-polarized lidar. A polarization lidar [11] could also improve specificity as it enables retrieval of the furriness on the bodies and glossiness of insect wings.

We have here presented a first-order model relating OCS and overtones to the flight heading or observation aspect. We have not distinguished between the pitch- and the altitude-angle of the velocity vector although these can be biased thus requiring spherical harmonics of odd degrees. Alternatively, if cataloged such discrepancy angle between pitch and altitude angle of velocity could be provided separately. In the present model we have not accounted for acceleration and have not distinguished insect self-propulsion from wind speed (which was zero in the laboratory settings presented). If required, models of higher accuracy accounting for such details, could be superimposed at later stage and treated as perturbation theory known from atomic physics, essentially following the line of arguments in this report. Since the presented model does not account for acceleration and wind the yaw- and azimuth angle of the velocity is not differentiated. It can be expected that the harmonic content depend on the roll-angle, however roll is zero without wind and acceleration, and for the application of estimation insect fluxes in the landscape roll angle have limited value. For the application of correcting projected cross section the roll would have marginal influence on the DC component on the cylindrical thorax, marginal influence on the fundamental and low harmonics and somewhat higher influence on the specular components.

In summary, the proposed method could solve both the problem of deriving the absolute size of insects from their projected cross section, and at the same time yield statistical fluxes of populations in the landscape. We expect this approach to enable biologists to analyze flying fauna in novel, quantitative ways. Such data sets could for instance serve as indicators of ecosystem functioning and diversity, as well as be used for monitoring the status of economically important groups such as pollinators. Importantly, this approach will also enable the study of temporal distribution and flight patterns of disease vectors and pests[40], in combination with data from currently used methodologies where suction-, pheromone, and light-traps are employed. Furthermore, information on age distribution of insects will make it possible to predict densities and adjust management accordingly by minimal pesticide use through strategic use at the right moment in the pest’s life stage. Finally, these methods are not uniquely suited to the study of insects; similar approaches could be applied to birds, bats and oscillating organism in the aquatic fauna, shedding light on biological questions also underwater.

## Supporting Information

S1 File Compressed zip-archive containing complete data for reproducing all figures presented. (RAR)
